# Gut Microbiota: Cardiovascular Disease Prevention and Targeted Therapies

**DOI:** 10.3390/biomedicines14061210

**Published:** 2026-05-27

**Authors:** Monica Loguercio, Domenico Mario Giamundo, Alessia Giglio, Emanuela Buda, Marco Ambrosetti, Francesco Perone

**Affiliations:** 1 Cardiovascular Rehabilitation Unit, ASST Crema, Santa Marta Hospital, 26027 Rivolta D’Adda, Italy; msloguercio@gmail.com (M.L.); marco.ambrosetti@asst-crema.it (M.A.); 2 Department of Systems Medicine, Tor Vergata University, 00133 Rome, Italy; jamundus20@libero.it; 3 Cardiology Unit, Department of Clinical and Experimental Medicine, University of Messina, 98124 Messina, Italy; alessiagiglio90@gmail.com; 4 Department of Internal Medicine and Cardiology, University Hospital, 6020 Innsbruck, Austria; emanuelabuda92@gmail.com; 5 Cardiac Rehabilitation Unit, Rehabilitation Clinic ‘Villa delle Magnolie’, 81020 Castel Morrone, Caserta, Italy

**Keywords:** gut microbiota, cardiovascular disease, diet, exercise, enzyme inhibitors, fecal microbiota transplantation

## Abstract

The gut microbiota has emerged as a key regulator of cardiovascular health, influencing metabolic, inflammatory, and vascular pathways. Growing evidence indicates that gut dysbiosis, characterized by reduced microbial diversity, depletion of beneficial short-chain fatty acid–producing bacteria, and enrichment of pro-inflammatory taxa, is associated with major cardiovascular risk factors and disease progression. Microbial-derived metabolites, including trimethylamine/trimethylamine N-oxide, short-chain fatty acids, amino acids and bile acids, may play a central role in modulating lipid metabolism, endothelial function, inflammation, and thrombosis, although the underlying mechanisms remain incompletely understood. Recent multi-omics approaches have expanded this understanding by identifying personalized microbiome–metabolome signatures linked to cardiovascular risk, supporting a shift toward precision medicine. In this review, we summarize current evidence on the composition and functional role of the gut microbiota in cardiovascular disease and critically discuss emerging microbiota-targeted strategies. These include dietary interventions, prebiotics, probiotics, synbiotics, antibiotics, enzyme inhibitors, and fecal microbiota transplantation, which may contribute to both the prevention and adjunctive treatment of cardiovascular conditions. In addition, we address the challenges of integrating gut microbiota management into clinical practice and highlight the importance of tailored strategies, including exercise-based interventions, microbial enzyme inhibitors, and postbiotics. Despite promising preclinical and early clinical data, the translation of microbiome-based therapies into routine practice remains limited by heterogeneity in study design, the lack of standardized protocols, and incomplete mechanistic understanding. Overall, targeting the gut microbiota represents a novel and potentially complementary approach for cardiovascular disease prevention and management, warranting further well-designed clinical studies.

## 1. Introduction

The human body harbors a vast and diverse community of microorganisms—including bacteria, viruses, fungi, archaea, and protozoa—collectively referred to as the microbiota, which colonize multiple anatomical sites such as the skin, respiratory tract, genitourinary system, and gastrointestinal tract. Among these, the gut microbiota represents the most complex and densely populated microbial ecosystem [1]. It consists of a dynamic network of microorganisms, in which bacteria are the predominant component, but viruses, including bacteriophages, as well as fungi and archaea, also play important roles in shaping microbial interactions and ecosystem stability. Recent estimates suggest that the human gut contains tens of trillions of microorganisms (approximately 10^14^ bacterial cells, comprising up to ~2000 species), organized into highly interactive communities that are essential for maintaining host homeostasis [2]. The establishment of the gut microbiota begins at birth, with early colonization influenced by maternal and environmental microbial exposure. In the first few hours of life, the mother’s vaginal and fecal microbiomes are usually the most important sources of inoculum. During early life, factors such as mode of delivery, breastfeeding, and diet contribute to the progressive diversification and maturation of the gut microbiota, which generally reaches a relatively stable, adult-like configuration by approximately three years of age [3]. Disruptions of normal early-life microbial colonization, such as those associated with preterm birth, may lead to altered microbial trajectories and reduced microbial diversity. In this context, maternal microbial communities appear to play a critical role: for instance, vaginal dominance of *Lactobacillus crispatus* has been associated with protective antimicrobial activity against pathogens implicated in preterm birth, whereas its depletion may favor dysbiosis and increase the risk of adverse pregnancy outcomes. Overall, these findings suggest that early host–microbiota interactions may contribute to long-term health [4].

The gut microbial community is primarily dominated by the phyla *Bacteroidetes* (recently reclassified as Bacteroidota) and *Firmicutes* (recently reclassified as Bacillota), whose relative abundance has been widely explored as a potential marker of gut health. Within the intestine, these microorganisms support essential functions such as digestion, immune regulation, epithelial turnover, and metabolic homeostasis, influencing pathways related to energy balance and nutrient metabolism [2]. However, alterations in microbial composition and function, referred to as dysbiosis, have been associated with a wide range of pathological conditions. The impact of the gut microbiota depends not only on specific taxa but also on their abundance, functional capacity, and interaction with the host [2,5].

Microbiota composition is shaped by multiple factors, including genetics, age, host dietary habits and the gut milieu. Additionally, external influences like intense physical and psychological stress, sleep disturbances and disruptions to circadian rhythms can also play a significant role [6]. Dysbiosis has been associated with a wide range of pathological conditions, including obesity, malnutrition, inflammatory bowel diseases, neurological disorders, cancer, and cardiovascular disease (CVD) [7,8]. In the cardiovascular context, these microbial alterations have been linked to several conditions, including atherosclerosis, hypertension, heart failure (HF), thrombosis, dyslipidemia and insulin resistance [9]. Early studies demonstrated the presence of bacterial DNA within atherosclerotic plaques, suggesting a potential contribution of microbial components to vascular pathology [10]. More recent data support a mechanistic role of gut-derived factors in modulating inflammation, endothelial function, and cardiometabolic pathways [2].

A critical aspect of the gut–heart axis is the integrity and function of the gut barrier in relation to CVD. Under physiological conditions, the intestinal barrier is maintained by structural mechanisms such as tight junctions, mucus layer production, and immune regulation. However, in patients with HF and cardiometabolic conditions, the integrity of the intestinal barrier may be impaired [11,12], facilitating the translocation of microbial products such as lipopolysaccharide (LPS) into the circulation. This phenomenon, known as endotoxemia, triggers a pro-inflammatory response. Overall, gut barrier dysfunction is increasingly recognized as a contributing factor within the complex gut–heart axis, rather than a standalone mechanism, and may represent a promising therapeutic target.

Beyond compositional changes and intestinal barrier integrity, emerging evidence suggests that the gut microbiota may act as a metabolically active “endocrine-like” organ, modulating host physiology through microbiota-derived metabolites and signaling pathways; in this context, microbiota-derived metabolites and host–microbiome interactions are increasingly recognized as key mediators within the gut–heart axis [13].

Bile acids (BAs), synthesized in the liver and subsequently modified by the gut microbiota, have been implicated in the regulation of lipid metabolism, glucose homeostasis, and inflammatory pathways. Similarly, short-chain fatty acids (SCFAs), mainly produced through microbial fermentation of dietary fibers, may contribute to blood pressure regulation, myocardial repair, and immune modulation [2]. In contrast, trimethylamine-N-oxide (TMAO), a microbiota-derived metabolite, has been associated in several studies with increased thrombotic risk, atherosclerosis, and adverse cardiac remodeling, although its causal role remains to be fully elucidated [14]. Collectively, these findings highlight how the gut microbiota may contribute to cardiovascular health through multiple interconnected mechanisms. Emerging data further suggest that these interactions are complex, dynamic, and context-dependent, being influenced by the host’s metabolic and inflammatory milieu. However, since part of this evidence derives from preclinical studies or preprints, it should be interpreted with caution and requires confirmation in well-designed, peer-reviewed clinical investigations [15]. This article provides an overview of the field, focusing on recent advancements and suggesting potential preventive and therapeutic strategies.

## 2. Gut Microbiota and Pathological Pathways Involved in the Development of Cardiovascular Disease and Risk Factors

The gut microbiota is essential for preserving human health, significantly impacting physiological functions such as metabolism, immunity, and inflammation [2]. Consisting of a vast array of microbial species, including *Firmicutes*, *Bacteroidetes*, *Actinobacteria*, *Fusobacteria*, Proteobacteria, *Verrucomicrobia*, and *Cyanobacteria,* the gut microbiota encodes a significant number of functional genes that outnumber those of the human genome [16]. The gut microbiota functions as an “endocrine-like” organ, influencing complex pathways involved in CVD. Beyond local effects, it contributes to systemic inflammation and insulin resistance through immune and metabolic modulation, and may also promote atherosclerosis and thrombosis via microbiota-derived metabolites and effects on platelet reactivity and vascular function [2,13,17].

### 2.1. Alterations in the Composition of Intestinal Microbiota: Dysbiosis and Cardiovascular Disease and Risk Factors

Emerging evidence highlights a potential interplay between gut microbiota composition and cardiovascular health, suggesting that several cardiovascular diseases could be associated with gut microbiota dysbiosis (Table 1).

Early studies investigating microbial signatures in coronary artery disease (CAD) used 16S rRNA gene sequencing to identify bacterial taxa associated with atherosclerotic plaques and alterations in the oral and gut microbiota [10]. Subsequent research reported distinct gut microbial changes in CAD patients, including increased levels of *Lactobacillus* spp. (phylum *Firmicutes*) and a reduction in *Bacteroides* and *Prevotella* spp. [18]. More recent metagenomic studies have further characterized microbial signatures associated with atherosclerosis and CAD, showing an increased abundance of taxa such as Enterobacteriaceae and oral-derived bacteria including *Streptococcus* and *Veillonella*, and *Collinsella*, which have been linked to pro-inflammatory pathways and altered lipid metabolism. Conversely, a consistent reduction in beneficial butyrate-producing bacteria, including *Faecalibacterium*, *Roseburia*, and *Eubacterium*, has been reported. These taxa produce SCFAs, metabolites known for their anti-inflammatory and vasculoprotective effects, suggesting that their depletion may contribute to atherosclerotic processes [13,19].

HF has also been associated with alterations in gut microbial composition. Several studies suggest a reduction in microbial diversity and a relative depletion of core commensal bacteria in these patients compared with healthy controls [20]. These changes may include a decreased abundance of beneficial SCFA-producing bacteria, and a relative enrichment of opportunistic, potentially pro-inflammatory taxa, such as *Escherichia*, *Klebsiella*, *Streptococcus*, and *Enterococcus*. In more advanced stages of HF, features of dysbiosis may also extend to include other microbial species, such as *Campylobacter* and *Candida* spp., although findings remain heterogeneous and should be interpreted with caution [12].

Early studies have shown that type 2 diabetes mellitus (T2DM) is associated with gut dysbiosis, including reduced butyrate-producing bacteria (*Faecalibacterium*, *Roseburia*) and increased pro-inflammatory taxa such as *Lactobacillus*, *Streptococcus*, and Proteobacteria [21]. Furthermore, insulin resistance has also been linked to elevated branched-chain amino acids associated with specific microbes, including *Prevotella copri* and *Bacteroides vulgatus* [22], supporting a possible role of the microbiome–metabolome axis in cardiometabolic disease.

Hypertension, a major contributor to CVD, has been linked to high dietary salt intake. Preclinical evidence suggests that a high-salt diet may alter gut microbiota composition, notably reducing *Lactobacillus murinus* and promoting TH17-mediated inflammation, contributing to salt-sensitive hypertension; these effects were partially reversed by *Lactobacillus* supplementation [23].

More recent multi-omics analyses have further expanded these observations, suggesting that personalized microbiome–metabolome signatures may be associated with coronary artery disease risk, with specific microbial taxa and derived metabolites potentially contributing to alterations in lipid metabolism, inflammatory pathways, and cardiometabolic risk profiles [24]. However, a major challenge in this field remains the distinction between causality and association within the gut–heart axis. Most available human studies are observational and cross-sectional, thereby limiting the ability to determine whether gut microbiota alterations act as causal drivers of cardiovascular disease or instead reflect secondary changes related to diet, pharmacological treatments, systemic inflammation, or coexisting comorbidities. While experimental studies in animal models support a potential causal role for specific microbial metabolites and pathways, the translation of these findings to humans remains limited. Moreover, reverse causation and residual confounding cannot be excluded in many clinical studies. Therefore, current evidence should be interpreted with caution, and the contribution of gut microbiota to cardiovascular disease should be considered within a complex, bidirectional framework rather than a strictly unidirectional causal relationship [9,15,19].

**Table 1:** Overview of the association between dysbiosis and cardiovascular diseases. T2DM, Type 2 Diabetes Mellitus. CAD, coronary artery disease.

	T2DM/Obesity	Hypertension	CAD/Atherosclerosis	Heart Failure
***Dysbiosis***	↑ *Lactobacillus, Streptococcus*, and Proteobacteria [20] ↓ *Roseburia and Faecalibacterium* [20]	↓ *Lactobacillus Murinus* [22]	↑ *Lactobacillus* (phylum *Firmicutes*) [17] ↓ *Bacteroides and Prevotella* spp. [17]	Bacterial overgrowth (*Escherichia*, *Klebsiella*, *Streptococcus*, and *Enterococcus*) [12]

### 2.2. Microbial Metabolites as Mediators

**Figure 1:**
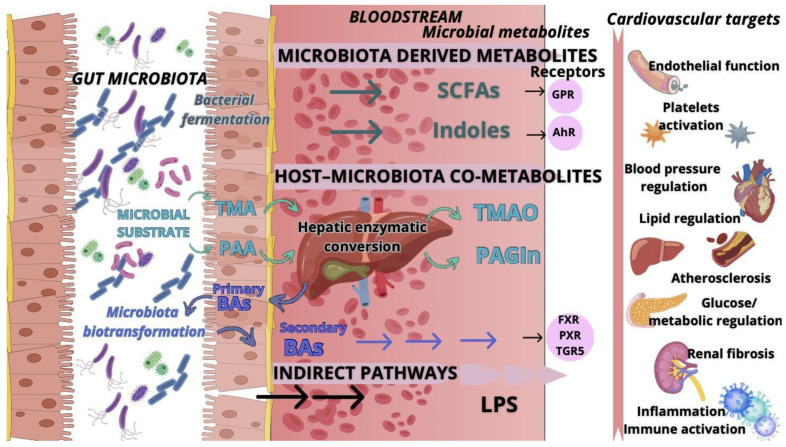
**Gut microbiota–host interactions and metabolic pathways involved in cardiovascular disease.** Gut microbial activity contributes to cardiovascular regulation through multiple, partly overlapping mechanisms that can be grouped into three main categories. (1) **Microbiota-derived metabolites**, including short-chain fatty acids (SCFAs) and indole derivatives, are produced through bacterial fermentation and generally exert anti-inflammatory, endothelial-protective, and metabolic regulatory effects. (2) **Host–microbiota co-metabolites**, generated through combined microbial and host metabolism, include trimethylamine N-oxide (TMAO), phenylacetylglutamine (PAGln) and bile acids (BAs). These metabolites are involved in lipid handling, vascular tone regulation, thrombosis, and cardiometabolic signaling and have been linked to pro-atherogenic and pro-thrombotic pathways. (3) **Indirect pathways**, driven by gut barrier dysfunction and increased permeability, allow translocation of microbial components such as lipopolysaccharide (LPS), triggering systemic inflammation, immune activation, and endothelial dysfunction. Together, these interconnected mechanisms influence lipid metabolism, vascular inflammation, immune responses, and atherosclerotic progression. Most evidence derives from preclinical and observational human studies, and the causal contribution of these pathways to cardiovascular disease remains to be fully established. Bas, bile acids. LPS, lipopolysaccharide. PAA: phenylalanine. PAGIn: phenylacetylglutamine. SCAFAs, short-chain fatty acids. TMA: trimethylamine. TMAO, trimethylamine N-oxide.

In addition to dysbiosis and direct alterations in bacterial patterns, increasing evidence suggests that the gut microbiota can also influence host processes through bioactive metabolites that can directly or indirectly affect distal organs. The gut microbiota may communicate with the host through multiple mechanisms mediated by microbially derived metabolites (Figure 1).

#### 2.2.1. SCFAs

In the intestinal environment, anaerobic fermentation of indigestible dietary fibers and complex carbohydrates, including resistant starch, pectin, inulin, and other polysaccharides, produces SCFAs, primarily acetate, propionate and butyrate. These metabolites are absorbed by the intestinal epithelium into the portal circulation, and supply roughly 5% to 10% of the host’s daily energy requirements. Once absorbed from the intestinal lumen, SCFAs reach the liver via the portal circulation and can subsequently enter the systemic circulation. Their biological effects are mainly mediated through the activation of G-protein-coupled receptors expressed on endothelial, immune, and adipose tissue cells, as well as through inhibition of histone deacetylases [25]. SCFAs are involved in various physiological functions, including immune regulation and metabolic processes across different tissues and organs. They may play a role in modulating immune functions such as chemotaxis and phagocytosis, promoting T-cell expansion, suppressing inflammatory signaling pathways and reducing the production of pro-inflammatory cytokines. Additionally, SCFAs help maintain intestinal barrier integrity, enhance host defense against pathogens, and contribute to the regulation of fluid and electrolyte absorption and the autonomic nervous system. The potentially anti-inflammatory properties may contribute to mitigating chronic inflammation and metabolic disorders associated with atherosclerosis, exerting potentially beneficial cardiovascular effects, including improvements in endothelial function, reductions in blood pressure, and attenuation of cardiac hypertrophy and fibrosis [2,13,25]. Individuals with T2DM exhibit alterations in gut microbiota composition, including a reduced presence of butyrate-producing bacteria [21], which are associated with impaired metabolic homeostasis. However, the causal relationships remain complex and bidirectional. Similarly, clinical studies in HF patients have reported a decreased presence of SCFA-producing bacteria, while higher circulating levels of SCFAs, particularly propionate and butyrate, have been associated with improved cardiac function [26].

#### 2.2.2. Bile Acids

Bile acids (BAs) are synthesized in the liver from cholesterol to form primary bile acids, which are secreted into the intestine to facilitate lipid digestion and the absorption of fat-soluble vitamins. In the gut, microbiota-mediated deconjugation and biotransformation convert primary bile acids into secondary bile acids, modifying the composition of the bile acid pool and influencing enterohepatic circulation [27]. Secondary BAs act as signaling mediators linking the gut microbiota to cardiovascular function through activation of nuclear and membrane receptors, including the farnesoid X receptor (FXR), Takeda G protein-coupled receptor 5 (TGR5), and pregnane X receptor (PXR). Experimental and translational studies suggest that FXR may regulate lipid and glucose metabolism and modulate TMAO production via the enzyme flavin monooxygenase 3 (FMO3), thereby influencing cholesterol homeostasis and inflammatory pathways. TGR5 activation, primarily by secondary BAs, has been associated with anti-inflammatory signaling, including inhibition of NF-κB, reduced macrophage activation and foam cell formation, and improved endothelial function. In contrast, PXR signaling has been implicated in pro-atherogenic mechanisms, potentially through increased lipoprotein levels and enhanced CD36-mediated lipid uptake in macrophages [2,13].

Overall, the cardiovascular effects of BAs appear to be context-dependent and influenced by their concentration and composition: physiological levels are generally associated with endothelial protection and metabolic regulation, whereas excessive accumulation, particularly of hydrophobic secondary BAs, has been linked to oxidative stress, endothelial dysfunction, and myocardial injury [25]. Alterations in BA metabolism, driven by gut microbiota activity, may therefore contribute to cardiometabolic diseases, including atherosclerosis, CAD, and HF, although many of these mechanisms are primarily supported by preclinical evidence.

#### 2.2.3. Amino Acid-Derived Microbial Metabolites

Dietary amino acids can be metabolized by both host and gut microbial enzymes, generating metabolites that may influence cardiovascular health. Among them, branched-chain amino acids (BCAAs)—leucine, isoleucine, and valine—have been associated with metabolic remodeling of the failing heart, particularly in heart failure with preserved ejection fraction (HFpEF), where impaired catabolism may promote myocardial hypertrophy and fibrosis [25]. Aromatic amino acids (AAAs), including phenylalanine, tryptophan, and tyrosine, are metabolized by the gut microbiota into bioactive compounds. For example, phenylalanine-derived phenylacetylglutamine (PAGln) has been associated with increased platelet reactivity and cardiovascular risk, potentially mediated, at least in part, through adrenergic receptor activation, and has also been linked to heart failure progression and atrial fibrillation, although its predictive role remains uncertain [26]. Similarly, microbial catabolism of tryptophan and tyrosine generates metabolites that have been implicated in vascular dysfunction, particularly in chronic kidney disease [13]. Among these, indole derivatives such as indole-3-propionic acid (IPA) and indole-3-acetic acid (IAA) may exert beneficial cardiometabolic effects, including anti-inflammatory and antioxidant actions and improved vascular function, although their impact appears context-dependent and not yet conclusive [2,13,25].

#### 2.2.4. TMAO

One molecule, later identified as TMAO, has been suggested to play a key role in the association with CVD risk. TMAO, a proatherogenic metabolite, is produced when the gut microbiota metabolizes specific nutrients, such as choline, lecithin, and L-carnitine, which are found in high amounts in animal-based foods like red meat, fish, and dairy products. These compounds are broken down by specialized enzymes (TMA lyases), transcribed by the CutC/CutD gene cluster, in the gut, releasing trimethylamine (TMA) and acetyl aldehyde. TMA is then absorbed into the bloodstream and processed by the liver, where it is oxidized to TMAO, mainly by the enzyme FMO3 [14]. Experimental evidence from preclinical models suggests that TMAO may contribute to pro-atherogenic processes through multiple mechanisms, including the promotion of oxidative stress, modulation of macrophage activity and foam cell formation, alterations in cholesterol transport and lipid metabolism, enhanced low-density lipoprotein (LDL) modification, increased monocyte adhesion via upregulation of vascular adhesion molecules, and progression of atherosclerotic lesions [26]. Plasma TMAO levels have been reported to correlate with CAD in a dose-dependent manner in some observational studies, although this association does not necessarily imply causality and may be influenced by residual confounding [2,14]. Higher TMAO concentrations have also been associated with features of coronary plaque vulnerability, including instability, rupture, and the presence of non-culprit lesions [2]. Elevated TMAO levels have been linked to adverse clinical outcomes, including increased all-cause mortality, particularly in patients with chronic kidney disease, and have also been observed in both chronic and acute heart failure, where they correlate with worse prognosis, impaired renal function, and a higher risk of rehospitalization [26,28,29]. In some clinical settings, such as cohorts undergoing elective coronary angiography after acute myocardial infarction, higher plasma TMAO concentrations have been associated with an increased risk of major adverse cardiovascular events, including stroke, myocardial infarction, revascularization, and death [28]. Moreover, TMAO concentrations have been associated with markers of systemic inflammation (e.g., C-reactive protein and IL-1β) and immune activation, while experimental evidence suggests a potential role in enhancing platelet reactivity and thrombotic risk [30,31]. Importantly, elevated TMAO levels have been proposed as a potential predictor of cardiovascular risk; however, this association remains debated, particularly after adjustment for traditional risk factors such as blood pressure, cholesterol, and triglycerides. While TMAO may reflect underlying cardiometabolic and renal dysfunction, its independent prognostic value is not fully established. Evidence from observational studies suggests that the strength of the association between TMAO and cardiovascular outcomes often attenuates after adjustment for key clinical variables, including renal function, age, and comorbidities. Notably, renal function is a major determinant of circulating TMAO levels, as TMAO is primarily cleared by the kidneys; thus, elevated concentrations may partly reflect reduced clearance rather than a direct causal effect [9,11,14]. Furthermore, heterogeneity across clinical studies indicates that the prognostic value of TMAO may vary according to population characteristics and clinical context, which may explain the loss of statistical significance observed in some multivariable analyses [9]. Overall, while TMAO remains a promising biomarker and potential therapeutic target, its clinical utility and causal role require further validation in well-designed longitudinal and interventional studies.

## 3. Targeted Therapies

### 3.1. Diet

**Figure 2:**
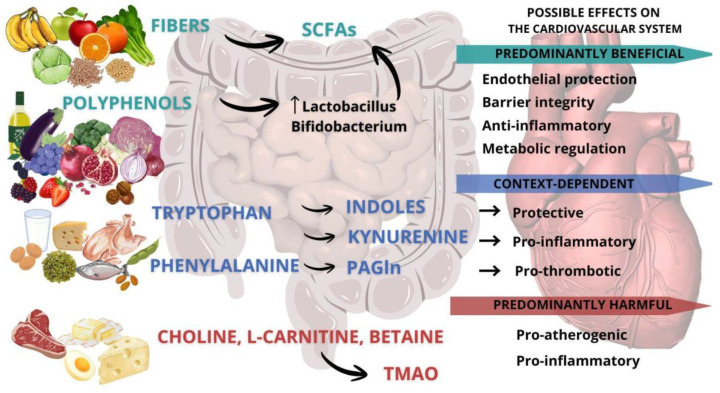
Impact of dietary patterns on gut microbiota and cardiovascular health. Dietary habits play a central role in shaping gut microbiota composition and function, thereby influencing cardiovascular risk. The Mediterranean diet, characterized by a high intake of fiber, polyphenols, and unsaturated fats, promotes microbial diversity and the production of beneficial metabolites such as short-chain fatty acids (SCFAs), which are associated with improved metabolic regulation, reduced inflammation, and enhanced endothelial function. In contrast, Western dietary patterns, typically rich in saturated fats and animal-derived nutrients (e.g., choline and L-carnitine), favor the growth of microbial taxa involved in the production of trimethylamine (TMA), leading to increased circulating levels of trimethylamine-N-oxide (TMAO), which has been linked to atherosclerosis and thrombosis. Microbial metabolism of dietary substrates also generates bioactive compounds such as phenylacetylglutamine (PAGln) and indole derivatives, which may exert either beneficial or detrimental cardiovascular effects depending on the context. Overall, dietary modulation of the gut microbiota represents a key mechanism linking nutrition to cardiovascular health; however, individual responses may vary, and causal relationships remain incompletely defined.

Several studies suggest that dietary interventions may be associated with a lower cardiovascular risk and could contribute to disease prevention; however, the available evidence remains largely observational and heterogeneous, and definitive causal relationships have yet to be firmly established [32]. The Mediterranean diet (MD) is a diet rich in vegetables, fruit, fiber and fish and provides moderate consumption of dairy products and red wine, along with limited consumption of red meat, favoring lean meats. MD has been associated with a lower prevalence of CVD and reduced mortality, potentially through improvements in insulin sensitivity, reductions in plasma cholesterol and LDL levels, and favorable modulation of gut microbiota composition; however, the strength and causality of these associations remain to be fully established (Figure 2). The microbiota associated with MD is characterized by increased biodiversity (namely an increased number of identified bacterial species) and this characteristic is termed “alpha diversity”. Soluble fiber can be fermented by the colonic microbiota to generate SCFAs, which contribute to glucose homeostasis, lipid metabolism, and blood pressure regulation and intestinal barrier integrity. SCFAs act through receptors such as GPR41, GPR43 and GPR109A in intestinal epithelial cells, promoting glucagon-like peptide 1 (GLP-1) secretion, and improving glucose homeostasis [25]. Additionally, OLFR78, expressed in vascular smooth muscle cells and the juxtaglomerular apparatus, contributes to blood pressure regulation via renin release with experimental studies suggesting that alterations in these pathways may affect blood pressure control [33].

Beyond fiber fermentation, various dietary bioactive compounds can influence gut microbiota composition. Polyphenols (e.g., resveratrol, gallic acid, and anthocyanins) promote beneficial taxa and may improve metabolic and inflammatory profiles, with experimental evidence also suggesting additional cardiometabolic effects, such as blood pressure modulation [34,35]. Gut microbes also convert dietary substrates into bioactive metabolites: tryptophan-derived indole metabolites may exert protective metabolic effects via aryl hydrocarbon receptor (AhR) activation, whereas kynurenine pathway, metabolites are more often linked to inflammation. Similarly, phenylalanine-derived PAGln has been associated with increased platelet reactivity and cardiovascular risk [36].

Conversely, Western dietary patterns are characterized by low fiber intake and high consumption of saturated fats and animal-derived nutrients such as phosphatidylcholine, choline and L-carnitine which are converted by the microbiota to TMA, which in turn is converted to TMAO. Some researchers have indicated that a fiber-deficient diet is associated with unfavorable cardiac remodeling, potentially driving the development of cardiac fibrosis and the onset of hypertension [37]. Overall, reducing the intake of red meat and choline-rich foods, such as egg yolks and liver, may help attenuate gut microbiota-dependent production of trimethylamine and its downstream metabolite TMAO, potentially contributing to lower cardiovascular risk [25].

Intermittent energy restriction diets (IER) consist of a variety of diet types, the most studied being intermittent fasting and time-restricted feeding. Time-restricted feeding in humans has been associated with reductions in blood pressure and cholesterol, as well as improvements in insulin sensitivity. In addition, an increase in Bacteroidetes has been observed during fasting, increasing the alpha diversity of the microbiota during feeding [38].

Despite these promising results, there is still much to be understood about the interaction between diets, microbiota, and CVD.

### 3.2. Exercise

Several studies suggest that physical activity can modulate gut microbiota composition and diversity in humans, potentially influencing cardiovascular health, although findings remain heterogeneous. Regular exercise is generally associated with increased microbial diversity (alpha diversity) and enrichment of beneficial taxa, particularly SCFA-producing bacteria [39]. Exercise may also induce compositional shifts, including changes in the *Firmicutes* and *Bacteroidetes* ratio, and promote the growth of genera such as *Lactobacillus* and *Bifidobacterium*. These adaptations have been associated with improved intestinal barrier integrity, reduced endotoxin translocation, and lower systemic inflammation, potentially contributing to better metabolic and cardiovascular health [40]. Athletes often exhibit distinct microbiota profiles characterized by higher microbial diversity and SCFA production, and combined training modalities (e.g., aerobic plus resistance exercise) may exert greater effects on microbial diversity than single exercise types [41]. Notably, experimental evidence also suggests that the gut microbiota may mediate the cardioprotective effects of exercise after myocardial infarction, as disruption of the microbiota (e.g., via antibiotics) attenuates exercise-induced improvements in cardiac remodeling and function [42].

However, the impact of exercise on the gut microbiota depends on multiple factors, including type, intensity, and duration. While low-to-moderate exercise appears to support microbiome homeostasis, high-intensity and prolonged exercise may increase inflammation and induce dysbiosis or intestinal permeability (“leaky gut”). Although higher-intensity exercise may have a stronger effect on microbial diversity compared to low-intensity activity, these benefits may be offset by potential adverse effects when exercise is excessive [41]. Additionally, host-related factors such as diet, fitness level, and baseline microbiota composition further modulate these responses. Overall, despite growing evidence linking physical activity, gut microbiota, and health, including associations with muscle strength and functional capacity in aging, current data remain limited and sometimes inconsistent, highlighting the need for well-designed studies to better clarify causal relationships and underlying mechanisms.

### 3.3. Probiotics, Prebiotics, and Synbiotics

**Figure 3:**
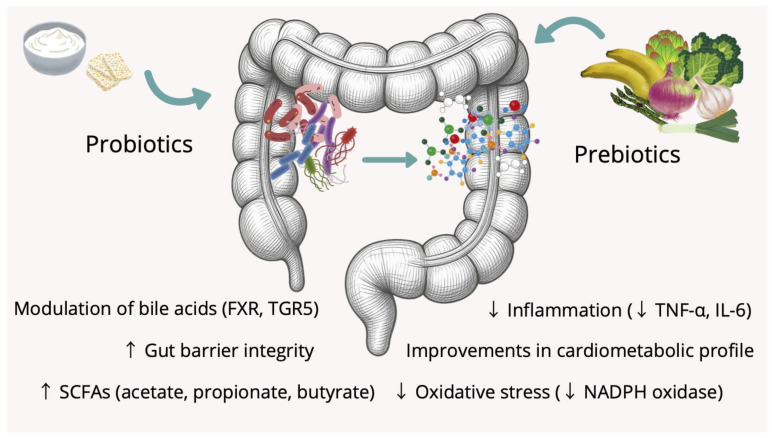
Possible effects of probiotics and prebiotics in the host. The figure summarizes the potential mechanisms by which probiotics and prebiotics modulate host physiology. These include regulation of bile acid metabolism through FXR (farnesoid X receptor) and TGR5 (Takeda G protein-coupled receptor 5), enhancement of gut barrier integrity, and increased production of short-chain fatty acids (SCFAs). Collectively, these effects may reduce systemic inflammation and oxidative stress, contributing to improvements in cardiometabolic profiles. SCFAs: short-chain fatty acids. FXR: Farnesoid X receptor. TGR5: Takeda G protein-coupled receptor 5.

Probiotics and prebiotics have been proposed as adjunctive strategies to modulate gut microbiota composition and metabolite profile in cardiovascular disease (Figure 3). Probiotics are live microorganisms found in a wide range of fermented products (such as yogurt, kefir and tempeh), commonly belonging to the genera *Lactobacillus* and *Bifidobacterium*, that may influence cardiometabolic regulation [43] through multiple mechanisms, including reinforcement of intestinal barrier integrity, modulation of immune responses, and alteration of microbial-derived metabolites [25]. Preclinical studies suggest that specific strains can reduce vascular oxidative stress, partly through downregulation of NADPH oxidase activity, and attenuate systemic inflammation in hypertensive models [44]. However, clinical studies and meta-analyses report only modest reductions in inflammatory markers (e.g., TNF-α and IL-6) [45] and small improvements in lipid parameters; overall, effect sizes are limited, and heterogeneity across strains, dosages, and study populations reduces reproducibility and precludes firm conclusions [46].

Prebiotics, such as galacto-oligosaccharides (GOS) and inulin-type fructans, are non-digestible substrates that selectively stimulate beneficial microbial taxa and enhance short-chain fatty acid production. Through increased SCFA availability and potential modulation of bile acid metabolism, prebiotics may influence glucose homeostasis, lipid metabolism, and inflammatory pathways. Nonetheless, most data derive from short-term interventions, and the clinical relevance of the observed metabolic changes remains uncertain [47].

Synbiotics, which combines probiotics and prebiotics, have emerged as a promising strategy to modulate gut microbiota composition and function, potentially enhancing short-chain fatty acid production and reducing inflammation [48].

Overall, although plausible biological mechanisms support a potential role for probiotics and prebiotics in cardiometabolic modulation, current evidence remains insufficient to justify their routine use in cardiovascular prevention [43]. These findings should be interpreted cautiously, as most data derive from small or preclinical studies, which often lack appropriate control groups and report heterogeneous results and inconsistent effects on key microbiota-derived metabolites linked to cardiovascular risk. In addition, marked inter-individual variability in gut microbiota composition likely contributes to heterogeneous responses, making it challenging to define optimal strains, dosages, and treatment durations [45].

In particular, interventions targeting the choline/carnitine–TMA–TMAO pathway have shown variable results, with several controlled studies reporting no significant reduction in circulating TMAO levels following probiotic or prebiotic supplementation [49]. Accordingly, large, well-designed randomized controlled trials are needed to clarify the efficacy and clinical applicability of these interventions. In this evolving context, research is increasingly shifting beyond conventional microbiota modulation toward more targeted and engineered approaches.

### 3.4. Next-Generation Microbiome-Based Therapies

Beyond conventional probiotics and prebiotics, recent research has focused on next-generation microbiome-based strategies aimed at achieving targeted modulation of microbial function. These approaches include genetically engineered bacteria, synthetic microbial consortia, and precision microbiome-editing techniques designed to selectively enhance beneficial metabolic pathways or inhibit harmful ones [50]. In particular, engineered microbial strains have been developed to reduce the production of pro-atherogenic metabolites such as trimethylamine (TMA) or to increase the synthesis of protective compounds, including short-chain fatty acids. The regulation of these mechanisms is closely involved in systemic inflammation, metabolic dysregulation, and adverse cardiac remodeling associated with myocardial infarction, as highlighted by a recent preprint study. Engineered bacteria are being explored as a means to modulate these pathways, potentially by reducing deleterious metabolite production and enhancing protective metabolic functions [15]. Unlike traditional probiotics, which often produce variable and strain-dependent effects, these strategies aim to achieve more predictable and mechanism-driven outcomes. Advances in synthetic biology have also enabled the design of microbial consortia with defined metabolic capabilities, potentially allowing personalized interventions tailored to individual microbiome profiles. However, these approaches remain largely experimental and are currently supported mainly by preclinical data. Important challenges include safety concerns, regulatory barriers, long-term stability of engineered strains, and potential off-target effects on host–microbiome interactions. Overall, next-generation microbiome-based therapies represent a promising but still emerging field, requiring further validation before clinical application in cardiovascular disease.

### 3.5. Antibiotics

Antibiotic-induced gut dysbiosis has emerged as an important factor influencing the gut–heart axis and cardiovascular health. Antibiotics can disrupt microbial diversity and composition, leading to depletion of beneficial taxa, reduced production of protective metabolites such as SCFAs, and alterations in bile acid metabolism. These changes may promote systemic inflammation, endothelial dysfunction, and metabolic disturbances, thereby contributing to cardiovascular risk. In addition, impairment of gut barrier integrity may facilitate endotoxin translocation, further exacerbating inflammatory responses [51].

The use of broad-spectrum antibiotics to modulate the gut microbiota as a strategy to reduce cardiovascular risk remains highly controversial. Although associations between atherosclerotic disease and specific pathogens have been reported, clinical trials have not demonstrated significant meaningful cardiovascular benefits from antibiotic therapy. Moreover, the potential cardiovascular effects observed in experimental models—particularly those related to blood pressure and metabolic pathways—have not been consistently replicated in clinical studies, with findings remaining heterogeneous and context-dependent, thereby limiting their translational applicability. Concerns related to adverse effects, disruption of microbial homeostasis, and the emergence of antibiotic resistance substantially limit their potential therapeutic application [9]. Additionally, the unpredictable recovery of the gut microbiota after antibiotic exposure and the lack of robust evidence supporting clinically relevant cardiovascular benefits highlight that antibiotics are unlikely to represent a viable long-term preventive strategy, particularly in light of the risks of antimicrobial resistance, and should therefore be reserved for the treatment of specific infections [9,51].

### 3.6. Non-Antibiotic Medications

Non-antibiotic pharmacological strategies targeting the gut microbiota represent a promising approach to modulate cardiovascular risk, as growing evidence highlights a bidirectional interaction between commonly used cardiovascular drugs and microbial composition; notably, inter-individual variability in the gut microbiota may influence drug pharmacokinetics and pharmacodynamics, contributing to heterogeneous therapeutic responses. Statins have been associated with microbiota-related modulation of bile acid metabolism and inflammatory pathways [52,53], whereas metformin has been shown to alter gut microbial composition, although the clinical implications of these changes remain under investigation [54].

Emerging evidence suggests that several cardiovascular drug classes, including angiotensin-converting enzyme (ACE) inhibitors, may interact with the gut microbiome and contribute to inter-individual variability in drug response, potentially contributing to improved cardiometabolic outcomes, although the underlying mechanisms remain to be fully elucidated [53]. In a recent clinical study involving patients with T2DM, empagliflozin was shown to reshape gut microbiota composition, promoting the selection of SCFA-producing bacteria while suppressing potentially harmful bacteria [55]. However, the extent to which these microbiota-related effects translate into clinically meaningful cardiovascular benefits remains to be fully elucidated.

### 3.7. Fecal Microbiota Transplantation

Fecal Microbiota Transplantation (FMT) is gaining recognition as a potential therapeutic intervention for addressing disorders related to gut microbiota imbalances. This procedure involves transferring bacteria from the feces of healthy donors into the intestines of patients to restore gut microbiota diversity and address specific conditions. Initially recognized for its effectiveness in treating recurrent *Clostridioides difficile* infections, FMT received its first FDA approval for this purpose in 2013. More recently, its potential application in CVD has gained interest, given the growing evidence linking gut microbiota to cardiovascular health [56]. Researchers are investigating how FMT could alter microbial composition to reduce metabolic and inflammatory risks associated with this condition [57]. Some studies have highlighted FMT’s potential to improve glycemic control [58], though findings are inconsistent. For instance, a meta-analysis reported only modest effects, including a slight increase in HDL cholesterol and minor improvements in insulin levels [59]. Other research has shown a higher prevalence of non-butyrate-producing Clostridiales in individuals with insulin resistance, with FMT leading to short-term improvements in insulin sensitivity and an increase in butyrate-producing bacteria. However, the effects of FMT appear variable over time, and long-term efficacy remains uncertain, likely reflecting both biological adaptation of the gut microbiota and host compensatory mechanisms, as well as heterogeneity in study design and treatment protocols [60,61]. Washed Microbiota Transplantation (WMT), a refined form of FMT, has shown preliminary and variable results in hypertensive patients demonstrating a short-term blood pressure-lowering effect [62]. This effect was particularly significant in patients who underwent WMT via the lower gastrointestinal tract and in those not previously on antihypertensive medications. Interestingly, the duration of the blood pressure-lowering effect from WMT was longer compared to conventional antihypertensive treatments. Recent pooled analyses indicate that WMT may improve lipid profiles, showing reductions in total cholesterol in the short term and in LDL-C and triglycerides over medium- to long-term follow-up, although no significant effects on HDL-C have been consistently observed. These effects are likely mediated by restoration of microbial diversity and increased abundance of SCFA-producing bacteria [61]. However, most evidence on fecal microbiota transplantation in cardiovascular and cardiometabolic conditions remains limited to small, heterogeneous studies, with inconsistent results and short-term follow-up. Therefore, its clinical applicability in cardiovascular disease is still uncertain and requires validation in large, controlled trials.

### 3.8. Enzyme Inhibitors/TMAO Inhibitors

Structural analogs of choline, such as 3,3-dimethyl-1-butanol (DMB), have been developed as targeted inhibitors of microbial TMA production. DMB specifically inhibits the activity of microbial TMA lyases. This competitive binding prevents choline from being cleaved into TMA, as DMB lacks the reactivity necessary for catalysis. Additionally, DMB may reduce the abundance of TMA-producing gut microbes, further lowering TMA production and its downstream metabolite, TMAO, which is implicated in cardiovascular risk factors like atherosclerosis and thrombosis [14,63]. This dual mechanism highlights DMB’s potential as a candidate therapeutic strategy for mitigating TMA production and its metabolic consequences. Acting as a non-lethal inhibitor, DMB significantly suppresses microbial TMA lyase activity without adversely affecting microbial growth. This characteristic makes it relatively non-toxic, enhancing its appeal as a treatment option. Moreover, DMB might naturally occur in certain foods, including balsamic vinegar, red wine, cold-pressed extra virgin olive oil, and grape seed oil, potentially providing a dietary source for reducing TMAO-related health risks [64]. More recently, a family of second-generation mechanism-based CutC/D inhibitors, such as iodomethylcholine (IMC) and fluoromethylcholine (FMC), has been developed with improved specificity and potency. These compounds exhibit significant antithrombotic effects in preclinical animal studies, reducing serum TMAO levels, platelet adhesion, aggregation, and carotid artery thrombus formation [65]. Unlike traditional bactericidal treatments, IMC and FMC are designed to be non-lethal to gut microbes, thereby minimizing risks of microbial resistance [66]. These inhibitors act selectively by irreversibly inhibiting choline TMA lyase after activation within the gut microbiota while avoiding systemic exposure [63,67]. Additionally, in preclinical models, they preserve essential host platelet and coagulation functions, suggesting a potentially safer alternative to conventional antiplatelet medications, with a lower bleeding risk [66,68]. Another strategy to reduce TMA could involve blocking the pathway further downstream at the liver by inhibiting FMO3. Reducing FMO3 expression has been shown to influence metabolic health, regulate cholesterol and glucose metabolism, reduce atherosclerosis, and improve metabolic profiles in mouse models [69]. However, inhibiting FMO3 may not be ideal, as it can lead to TMA accumulation, promoting inflammation and causing trimethylaminuria (“fishy odor syndrome”). Additionally, FMO3 is involved in the metabolism of several drugs, making it a less appealing target [67]. Importantly, these strategies are currently supported almost exclusively by preclinical evidence, and no large-scale human trials have yet confirmed their safety and efficacy. As such, their clinical translation remains at an early stage.

To better summarize the current evidence, microbiota-targeted interventions are categorized according to their proposed mechanisms, level of evidence, and key limitations (Table 2).

As shown in Table 2, while several strategies have demonstrated promising effects, most interventions remain limited by heterogeneous evidence and lack of large-scale clinical validation.

## 4. Gut Microbiota in Clinical Practice

In clinical practice, managing the gut microbiota requires a personalized approach, particularly in cardiovascular care. Personalized nutrition aims to modulate an individual’s microbiome to optimize dietary and metabolic responses, potentially enabling the design of tailored interventions based on microbiome composition, predicted responses, and the identification of beneficial foods. This concept is increasingly supported by multi-omics approaches, which enable a more precise characterization of individual microbiome profiles. A deeper understanding of the microbiome’s role in treatment responses is essential for advancing therapies and developing targeted approaches for cardiovascular health.

Research on the gut microbiome is increasingly focused on regulating dietary metabolites associated with CVD, particularly TMAO production. Studies are targeting non-essential components. Due to its possible correlation with cardiovascular risk, TMAO has been proposed as a potential biomarker for assessing the likelihood of atherosclerosis and related conditions. Promising strategies to reduce TMAO levels include dietary modulation, particularly reduction in TMAO precursors; however, approaches targeting host pathways require caution due to the essential role of nutrients such as choline. Blocking its absorption could lead to deficiencies and neurological issues. Therefore, increasing attention has been directed toward selectively targeting microbial TMA production in the gut without interfering with choline’s vital functions [67]. While broad-spectrum bactericidal antibiotics can transiently reduce circulating TMAO levels, their effects are not sustained and are limited by microbiota disruption and the risk of resistance. In contrast, targeted approaches such as non-lethal microbial enzyme inhibition have shown promise as alternative strategies for CVD modulation in preclinical models, reducing TMAO production and attenuating atherosclerosis, cardiac remodeling, and thrombosis without significantly altering overall microbiota composition and with minimal systemic side effects [64,65,67,70]. In animal models, second-generation inhibitors such as iodomethylcholine have been shown to reduce TMAO levels, leading to improved cardiac function by attenuating adverse remodeling and fibrosis in HF. These findings support the concept that targeting microbial enzymes involved in TMA production may represent a promising strategy to modulate host cholesterol and BA metabolism, and reduce cardiovascular risk [71]. Overall, microbiota-targeted inhibitors appear to be a promising therapeutic approach for cardiometabolic diseases, as well as in non-traditional and traditional cardiovascular risk factors [68], such as diabetes mellitus [72] and arterial hypertension [66], although evidence remains largely preclinical.

In contrast to pro-atherogenic metabolites such as TMAO, increasing attention has focused on beneficial microbiota-derived metabolites as therapeutic targets. Postbiotics are non-viable microbial products or components that confer health benefits without containing live microorganisms, thereby reducing safety concerns associated with probiotics. In particular, short-chain fatty acids (SCFAs), key components of postbiotics, modulate inflammation, endothelial function, and metabolic homeostasis, contributing to cardiovascular protection. Overall, postbiotics, especially SCFAs, represent a promising strategy for cardiovascular disease prevention and management, although further clinical validation is required [73].

Emerging evidence suggests that exercise may modulate gut microbiota composition, with structured training potentially associated with changes in microbial diversity and taxa linked to improvements in functional capacity and cardiometabolic parameters [74,75]. Experimental findings further indicate that the gut microbiome could partly mediate the beneficial effects of exercise on cardiac function; however, most of this evidence derives from preclinical models, and clinical data remain limited and sometimes inconsistent.

In clinical practice, assessment of the gut microbiota is still constrained by methodological challenges and marked inter-individual variability. Although distinct microbial patterns have been associated with cardiovascular risk and outcomes, supporting a potential role of the gut microbiome as a biomarker and therapeutic target [48,76], its use in predicting recurrent cardiovascular events remains preliminary, with current evidence suggesting possible associations between specific microbial and metabolic signatures and recurrence risk [77]. Overall, advances in multi-omics and precision medicine may facilitate the development of personalized microbiota-targeted strategies, although their clinical applicability requires further validation. 

### Multi-Omics Approaches and Precision Medicine

Advances in multi-omics technologies have significantly expanded the understanding of host–microbiome interactions in cardiovascular disease [78]. Integrative approaches combining metagenomics, metabolomics, transcriptomics, and proteomics allow a more comprehensive characterization of microbial composition and function, as well as their systemic effects on host physiology. These approaches have enabled the identification of individualized microbiome–metabolome signatures associated with cardiovascular risk, disease progression, and response to therapeutic interventions [79]. In particular, integration of microbial and metabolic profiling may provide insights into patient-specific pathways, supporting the development of precision medicine strategies [80]. Despite these promising advances, several limitations currently hinder clinical implementation. These include high costs, methodological heterogeneity, lack of standardized analytical pipelines, and challenges in data integration and interpretation. In addition, most available evidence derives from observational studies, and the clinical utility of multi-omics-based stratification remains to be validated in prospective trials. Overall, multi-omics approaches represent a key step toward personalized microbiome-based interventions; however, further research is required to translate these findings into routine cardiovascular clinical practice.

## 5. Conclusions

The gut microbiome plays a critical role in human health, influencing various physiological functions, including metabolism and immune responses. Imbalances, or dysbiosis, have been linked to several diseases, particularly cardiovascular conditions. Advances in understanding microbial pathways have opened new therapeutic avenues, focused on targeting gut bacteria to manage cardiovascular health. Personalized strategies hold promise for disease prevention and treatment, marking the microbiome as a key element in the future of precision medicine for CVDs. The non-reactive and targeted nature of gut microbiota-targeting inhibitors makes them a promising therapeutic avenue for modulating microbiota-related pathways in cardiovascular and metabolic diseases. While still in preclinical stages, ongoing studies are advancing toward human trials, offering hope for the transition of TMAO-lowering treatments from research to clinical applications. These developments highlight the potential for microbial-targeting therapies to play a significant role in managing cardiovascular health. Finally, emerging technologies like genetic engineering and nanotechnology offer promising tools to manipulate the gut microbiota, with the goal of improving health, especially in the context of CVD. These approaches could provide innovative solutions to manage and treat CVD by focusing on the gut microbiota’s role in disease processes.

## Figures and Tables

**Table 2:** Microbiota-targeted interventions in cardiovascular disease: mechanisms, level of evidence, and limitations.

Intervention	Proposed Mechanisms	Level of Evidence	Key Limitations
Mediterranean diet	SCFA production, reduced TMAO precursors, anti-inflammatory effects	Clinical (observational + RCTs)	Adherence variability, indirect effects
Exercise	Increased microbial diversity, SCFA production, improved gut barrier	Clinical + preclinical	Heterogeneity in protocols and populations
Probiotics/Prebiotics/Synbiotics	Modulation of microbial composition, SCFAs, bile acids	Mixed (small RCTs + preclinical)	Strain specificity, inconsistent results
Antibiotics	Reduction in microbial metabolites, altered microbiota composition	Preclinical + limited clinical	Resistance, dysbiosis, lack of long-term benefit
Non-antibiotic drugs (e.g., metformin, statins)	Indirect microbiota modulation, metabolic effects	Clinical	Mechanisms not fully understood
Fecal microbiota transplantation (FMT)	Restoration of microbial diversity, metabolic modulation	Limited clinical	Variability, safety concerns, short-term effects
Enzyme inhibitors (e.g., DMB, IMC)	Inhibition of TMA production → reduced TMAO	Preclinical	Lack of human trials
Next-generation microbiome therapies	Engineered microbes, targeted metabolic modulation	Preclinical	Safety, regulation, stability

## Data Availability

No new data were created or analyzed in this study.

## Abbreviations

The following abbreviations are used in this manuscript:

AAAs	Aromatic amino acids
ACE	Angiotensin-converting enzyme
AhR	Aryl hydrocarbon receptor
AR	Adrenergic receptors
BAs	Bile acids
BCAAs	Branched-chain amino acids
CAD	Coronary artery disease
CVD	Cardiovascular disease
DMB	Dimethyl-1-butanol
FMC	Fluoromethylcholine
FMO3	Flavin monooxygenase 3
FMT	Fecal Microbiota Transplantation
FXR	Farnesoid X receptor
HF	Heart failure
HFpEF	Heart Failure with preserved Ejection Fraction
IMC	Iodomethylcholine
GLP-1	Glucagon-like peptide 1
GOS	Galacto-oligosaccharides
HDL	High Density Lipoprotein
IAA	Indole-3-acetic acid
IER	Intermittent energy restriction diets
IPA	Indole-3-propionic acid
LDL	Low-Density Lipoprotein
LPS	Lipopolysaccharide
MD	Mediterranean diet
PAA	Phenylalanine
PAGln	Phenylacetylglutamine
PXR	Pregnane X receptor
SCFAs	Short-chain fatty acids
T2DM	Type 2 diabetes mellitus
TGR5	Takeda G protein-coupled receptor 5
TMA	Trimethylamine
TMAO	Trimethylamine-N-oxide
WMT	Washed Microbiota Transplantation
